# Controlled Release of Volatile Antimicrobial Compounds from Mesoporous Silica Nanocarriers for Active Food Packaging Applications

**DOI:** 10.3390/ijms23137032

**Published:** 2022-06-24

**Authors:** Tina Gulin-Sarfraz, Georgios N. Kalantzopoulos, John-Erik Haugen, Lars Axelsson, Hilde Raanaas Kolstad, Jawad Sarfraz

**Affiliations:** 1 Nofima-Norwegian Institute of Food, Fisheries and Aquaculture Research, P.O. Box 210, NO-1431 Ås, Norway; john-erik.haugen@nofima.no (J.-E.H.); lars.axelsson@nofima.no (L.A.); 2 Center for Materials Science and Nanotechnology (SMN), Department of Chemistry, University of Oslo, P.O. Box 1033, Blindern, NO-0315 Oslo, Norway; georgios.kalantzopoulos@kjemi.uio.no; 3 Imaging Centre, Faculty of Biosciences, Norwegian University of Life Sciences (NMBU), P.O. Box 5003, NO-1432 Ås, Norway; hilde.kolstad@nmbu.no

**Keywords:** mesoporous silica particles, nanocarriers, encapsulation, essential oil components, stimulus activated release, active packaging, antimicrobial packaging, green packaging

## Abstract

Essential oils and their active components have been extensively reported in the literature for their efficient antimicrobial, antioxidant and antifungal properties. However, the sensitivity of these volatile compounds towards heat, oxygen and light limits their usage in real food packaging applications. The encapsulation of these compounds into inorganic nanocarriers, such as nanoclays, has been shown to prolong the release and protect the compounds from harsh processing conditions. Nevertheless, these systems have limited shelf stability, and the release is of limited control. Thus, this study presents a mesoporous silica nanocarrier with a high surface area and well-ordered protective pore structure for loading large amounts of natural active compounds (up to 500 mg/g). The presented loaded nanocarriers are shelf-stable with a very slow initial release which levels out at 50% retention of the encapsulated compounds after 2 months. By the addition of simulated drip-loss from chicken, the release of the compounds is activated and gives an antimicrobial effect, which is demonstrated on the foodborne spoilage bacteria *Brochothrix* *thermosphacta* and the potentially pathogenic bacteria *Escherichia coli*. When the release of the active compounds is activated, a ≥4-log reduction in the growth of *B. thermosphacta* and a 2-log reduction of *E. coli* is obtained, after only one hour of incubation. During the same one-hour incubation period the dry nanocarriers gave a negligible inhibitory effect. By using the proposed nanocarrier system, which is activated by the food product itself, increased availability of the natural antimicrobial compounds is expected, with a subsequent controlled antimicrobial effect.

## 1. Introduction 

Growing consumer demand for safe food products with fewer synthetic preservatives has resulted in a search for alternatives from natural sources to improve the microbial safety of food. Natural additives are found in a wide variety of plants, and novel preservation methods are explored using these natural antimicrobials [1]. Essential oils are secondary metabolites from plants, composed of a mixture of volatile compounds, which have been widely studied for their antimicrobial, antioxidant, and antifungal properties [2,3,4,5]. The antimicrobial activity of essential oils is attributed to several mechanisms, mainly membrane disruption which increases permeability and subsequently impairs important cell functions [6,7]. The delivery mode of these natural antimicrobials is important for the resulting antimicrobial activity. When the antimicrobial substances are added using a time-release approach, lower concentrations can achieve the same inhibitory effect as in a direct addition approach [8]. Wang et al. have demonstrated a significantly larger antimicrobial effect of the active compound thymol when it was continuously added compared to instantly added, even if the final amount of thymol was the same [9].

These volatile active compounds have been studied both as direct additives to food and as food-packaging incorporated material. Some of these compounds have shown stronger antimicrobial effects in the vapor phase than in the liquid phase [10,11], which makes them good candidates as non-contact preservatives for active food packaging. The function of active food packaging systems is to prolong the shelf-life or to maintain or improve the condition of packaged food, by releasing or absorbing substances into or from the food or the environment surrounding the food [12]. There are several different concepts of active packaging; moisture absorbers, oxygen and ethylene scavengers, carbon dioxide emitters, as well as systems for actively releasing antimicrobial or antioxidant compounds. In antimicrobial active packaging, the active compounds can be directly incorporated into the polymer matrix, coated onto the packaging surface, or immobilized in sachets [13,14].

However, due to the sensitivity of the volatile compounds, to heat, oxygen and light, these substances can usually not resist the harsh processing conditions of conventional methods, such as extrusion or injection molding, which limits their incorporation into the polymer matrix. Coating techniques are not either beneficial in terms of a fast release of the volatile compounds, and there is also difficulty in mixing the substances into the coating formulation [15]. Thus, the application of nanotechnology has improved the usage of volatile natural compounds through encapsulation [16]. Originally, nanotechnology was recognized as a promising enabler for improving material properties, such as barrier and mechanical properties [17]. Later, the technology rapidly expanded to numerous applications within active and intelligent food packaging [18,19,20,21,22]. Encapsulation by inorganic nanocarrier matrices has lately received considerable attention in research. However, when incorporating loaded nanocarriers into the polymer matrix, the modification of the polymer matrix by the nanocarriers must be considered. Since sachets and pads are widely used for active food packaging and might be one of the most successful applications at the commercial level [23], these are potential holders for the loaded nanocarriers. When choosing a suitable nanocarrier, it is important to note that only nanoparticles covered by EU commission regulation No 10/2011 [24] are authorized for food packaging applications in the EU. These regulations apply both to nanoparticles directly in contact with food, and to nanoparticles used behind a functional barrier. The approved nanoparticles are silica, carbon black and titanium nitride. In addition, silicic acid is also approved, which is the by-product of dissolved silica. Hence, porous silica nanoparticles are the most suitable carriers for potential food packaging applications. Due to the vast design options for porous silica, the pore structure, surface area and surface chemistry can be tailored towards the properties of the compound to be encapsulated. These features are already well-studied in the biomedical field, for example, for drug delivery [25,26,27], diagnostic imaging [28,29], and for the stabilization of bioactive proteins [30].

Previous studies have reported that encapsulation of volatile compounds into inorganic nanocarriers can prolong the release of the compounds and thus enhance the antimicrobial effect by increasing the time the volatile compounds are present in the headspace [31,32,33]. However, we have earlier shown that the design of the nanocarrier highly influences the loading degree, the retention, and the subsequent release [34]. The encapsulation of thymol into mesoporous silica particles was efficient and only 10% of the loaded thymol amount was released from the mesopores in ambient conditions during a period of 7 days, while 90% of free thymol volatilized. Further, the encapsulated thymol could be released by water activation. Han et al. [35] have seen similar release behavior by mixing essential oils with hydrophilic starch beads directly in a sachet. The beads adsorbed the essential oil components and did not release them within a period of 30 min. However, when the starch was exposed to water the active components were released. Maia et al. [36] have also encapsulated thyme essential oil into starch to modulate the release by swelling the starch matrix in water.

According to Otoni et al. [23], the development of novel dual-action sachets is encouraged in order to further improve the efficiency of emitting sachets and absorbent pads. The dual-action approach is based on the removal of moisture or drip loss, and a simultaneous release of antimicrobial substances. The future intended use of the nanocarriers presented in this study is to be incorporated into absorbent pads, for a synergistic effect. The mesoporous silica particles presented in this study are specifically optimized for the encapsulation of volatile natural compounds. Due to the high surface area and optimal pore size, the nanocarriers can carry a high amount of cargo and further deliver a slow release where only half of the loading is released after 2 months. However, the release can be activated by simulated drip-loss from meat products, with a subsequent rapid increase of the volatile compounds in the headspace, and an enhanced antimicrobial effect. To the best of our knowledge, this is the first report on the long-term vaporization rate of essential oil components from mesoporous silica nanocarriers, with a simulated drip-loss activated burst release, which results in a rapid antimicrobial effect in the vapor phase on foodborne bacteria.

## 2. Results and Discussion

**Figure 1:**
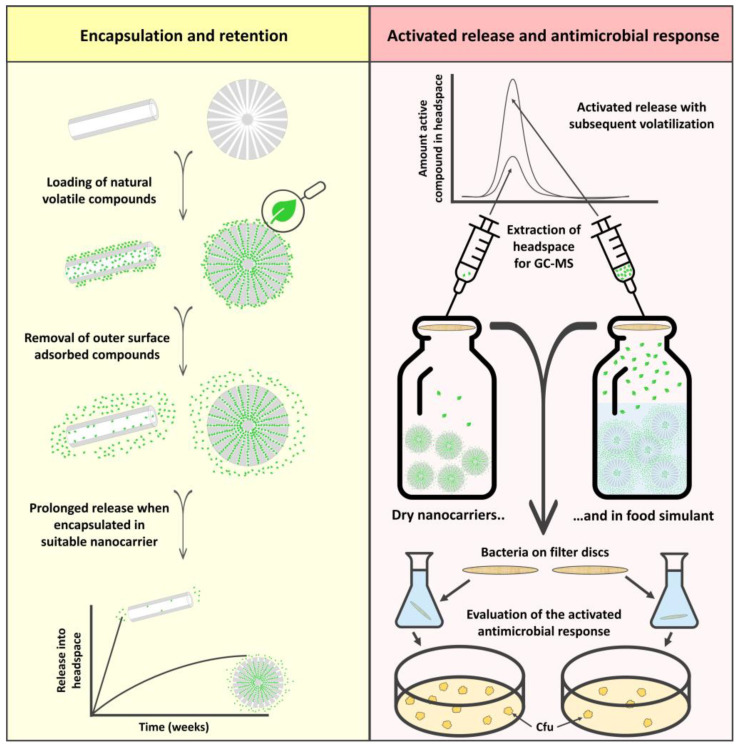
Schematic illustration of the study. The left (yellow) column represents the encapsulation of different volatile essential oil compounds into porous silica nanocarriers and halloysite nanotubes. A suitable nanocarrier gives rise to a sustained release profile which levels out after several weeks. The right (red) column demonstrates the activated release from the nanocarriers, and the resultant higher amount of active compounds in the headspace. The antimicrobial effect of the active compounds vaporized into headspace is investigated in closed containers, as schematically described in the figure.

The rationale of this work was to extend the use of natural antimicrobial compounds, such as essential oils and their active components, for food packaging applications by designing a suitable nanocarrier for these specific compounds. Nanocarriers are identified as carrier matrices with any external dimension in the nanoscale (1–100 nm) or those having internal or surface structures in the nanoscale [18]. We have earlier reported on the important aspect of choosing the right nanocarrier for specific active compounds [34]. With that in mind, we have here synthesized porous silica particles with a large surface area and a protective pore environment. Our investigation included the encapsulation of pure essential oil and different essential oil components. The retention of the compounds in the nanocarriers was further explored over a period of two months. Here, the well-studied and commercially available halloysite nanotubes were used in parallel for comparison purposes. The release of the compounds in simulated drip-loss from chicken was demonstrated, with the subsequent extraction of the headspace for GC-MS analysis. The antimicrobial response of the loaded nanocarriers was investigated in the vapor phase by activating the release of the active compounds. A schematic overview of the study is presented in Figure 1.

### 2.1. Characterization of the Nanocarriers

Mesoporous silica particles of type MCM-41 (Mobil Composition of Matter No. 41) [37] have received vast attention in various fields, due to their unique properties, such as high specific surface area and pore volume, and tunable pore sizes with a narrow pore size distribution. Typically, MCM-41 is produced by employing a basic aqueous solution of quaternary ammonium surfactants at elevated temperatures for the hydrolysis and condensation of the silica precursor. This material exhibits a hexagonal arrangement of uniform mesopores. Later, the addition of an alcohol as a co-solvent has been shown to allow for the control of the morphology of the material, thus leading to spherical MCM-41 particles [38,39]. 

**Figure 2:**
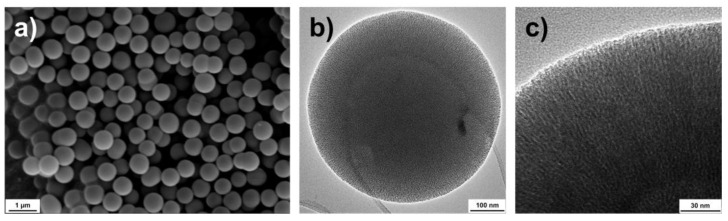
Scanning electron microscopy (**a**) and transmission electron microscopy (**b**,**c**) images of the spherical monodispersed nanocarriers.

**Figure 3:**
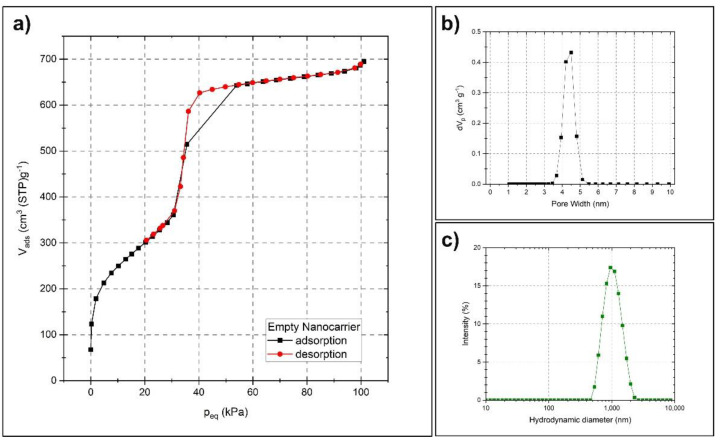
Characterization of the nanocarriers by nitrogen sorption and dynamic light scattering. Nitrogen physisorption isotherm (**a**), pore size distribution plot (**b**), and hydrodynamic size distribution of the particles (**c**).

For preparing a suitable carrier matrix for the volatile active compounds, an MCM-41 type synthesis was employed, which renders spherical particles sized around 600 nm. The scanning and transmission electron microscopy images (SEM and TEM) presented in Figure 2 reveal the spherical shape and the porosity of the monodispersed particles. The TEM images indicate that the pores might be distributed in a radially aligned direction. Pauwels et al. [40] performed a detailed study on the pore structure of spherical MCM-41 particles synthesized in the presence of ethanol and revealed a more complex pore order compared to standard MCM-41 materials. They found that the particles have a spherically symmetric pore distribution, starting from the inner part of the sphere and extending out to the outer surface. Only on a very local scale, the pores were distributed in a hexagonal array with their closest neighboring pores. Their findings correspond well with the TEM characterization of the particles presented here. To further determine the specific surface area, pore size and pore volume of the synthesized nanocarriers, nitrogen sorption measurement was utilized. Figure 3a presents a type IV isotherm (IUPAC nomenclature), which is typical for mesoporous materials. A specific surface area of 1120 m^2^/g, a pore size of 4.5 nm and a pore volume of 1.2 cm^3^/g were obtained using the Brunauer–Emmett–Teller (BET) and non-local density functional theory (NLDFT) methods. Figure 3b demonstrates the narrow pore size distribution.

The uniform size of the particles, as well as their dispersibility in aqueous media, was further investigated by dynamic light scattering (DLS). Figure 3c shows the narrow hydrodynamic size peak centered around 1000 nm, with a polydispersity index (PdI) of 0.14. The difference between the size determined by DLS and by SEM is a known phenomenon and is due to the different principles behind the measurement techniques, where DLS, in general, gives a larger particle size [41,42,43,44]. DLS determines the size in terms of their average hydrodynamic diameter by investigating their diffusive behavior by the light scattered from the particles in a liquid media. The diameter and the shape of the dried particles are directly measured from SEM images. However, these techniques are good complements for determining both the size, shape and dispersibility in aqueous media. The dispersibility of the particles in aqueous media is largely reflected by the zeta potential of the particles, where usually an absolute value of 30 mV is considered to provide good stabilization. The particles presented here measured a zeta potential of −20 mV in an aqueous solution (around pH 7), which might be sufficient to electrostatically stabilize a particle dispersion at least on shorter time scales [45].

### 2.2. Encapsulation of Natural Active Compounds

Due to the large surface area and high pore volume of these nanocarriers, they could carry a large amount of volatile active compounds. Four well-studied antimicrobial compounds; thymol [46], carvacrol [47], eugenol [48] and citral [49], were chosen for the study, as well as one essential oil; cinnamon oil [50]. The active compounds were efficiently loaded into the silica nanocarrier by the non-polar organic solvent cyclohexane. Mattos et al. [51] evaluated the affinity of thymol with silica support in thin layer chromatography by various solvents. They emphasized the need for loading thymol into the silica matrix using a suitable non-polar solvent, to promote spontaneous loading. It was further discussed that since thymol can be dissolved by the non-polar organic solvent, the cohesive crystal lattice energy of the compound that limits the dissolution is controlled, which subsequently assists in a favorable release. Following the loading, the nanocarriers were kept under a continuous vacuum for several hours to remove any active compound adsorbed onto the outer surface.

Given that the active compounds have a strong affinity to polar solvents [51], the compounds were eluted from the nanocarriers by ethanol to further determine the total encapsulated amount by spectroscopic methods. UV-Vis spectroscopy is a useful technique for the identification of pure compounds. Thymol, carvacrol and eugenol consist of only one UV-active molecular structure and can thus successfully be determined with this method. Citral, on the other hand, consists of two isomers named neral (*cis* isomer) and geranial (*trans* isomer), with overlapping UV spectra [52]. Cinnamon oil also consists of several compounds with overlapping UV-absorbance spectra, where eugenol is the most abundant one. However, both citral and cinnamon oil gave rise to only one UV-absorbance band when dissolved in ethanol, with a subsequent concentration-dependent calibration curve. Thus, for internal comparison of the loading and release, the UV-absorbance method was applied.

Additionally, the amount of loaded cargo was determined with thermogravimetric analysis (TGA). The percent active compound in the silica nanocarriers, as presented in Table 1, was calculated according to a previously reported method [53]. The loading degree of the nanocarriers was also compared to the commercially available halloysite nanotubes, and in accordance with our previous observations [34], the specific surface area and the pore structure of the nanocarriers play a significant role in the containment of all the active compounds. The halloysite nanotubes, which do not have a high surface area or mesoporous pore environment, could only load up to a tenth of the amount of the active compounds encapsulated into the silica nanocarriers (Supplementary Figure S1). 

### 2.3. Prolonged Release of the Incorporated Active Compounds

**Figure 4:**
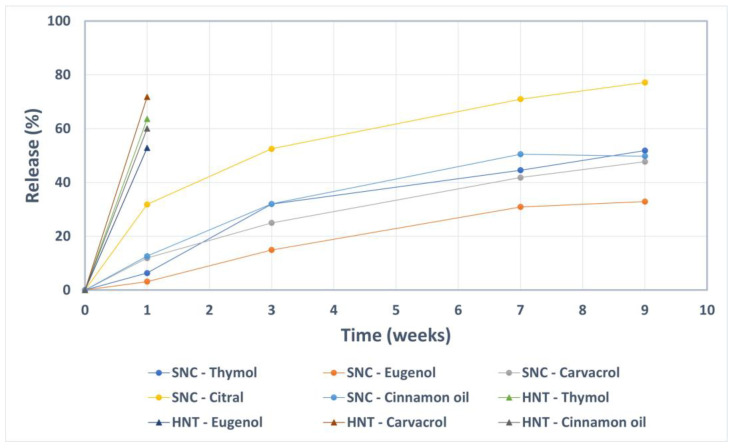
Prolonged release of the active compounds encapsulated into silica nanocarriers (SNC) compared to the control nanocarriers (halloysite nanotubes, HNT).

The release (in terms of vaporization) of the active compounds from the loaded nanocarriers was studied over a 9-week period in ambient conditions. As a control, halloysite nanotubes loaded with the same compounds were utilized. The results presented in Figure 4 reveal a slow vaporization of the active components from the silica nanocarriers. During the first week, the nanocarriers released only about 10% or less of the active compounds, except for citral where the released amount was 30%. The considerably higher vapor pressure of citral compared to the other compounds (Table 2) is probably the reason for the elevated vaporization rate. After 9 weeks, the silica nanocarriers still retained at least 50% of the loaded compounds (except for citral). On the contrary, after only one week, the control nanocarriers (halloysite nanotubes) lost an amount of 50–70% of the initial loading of thymol, carvacrol, eugenol and cinnamon oil. For these, the retained amount citral was under the detection limit after one week.

**Figure 5:**
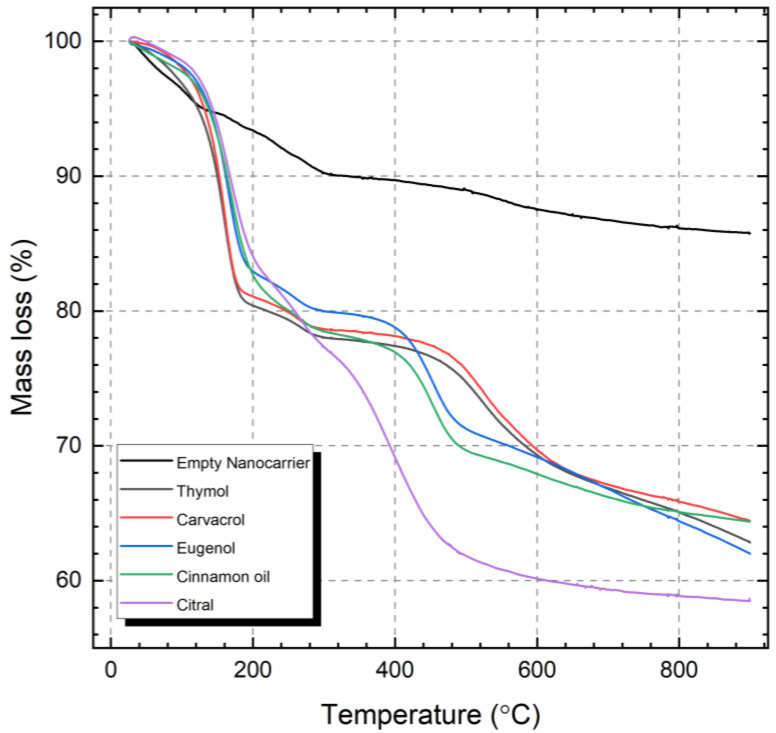
Thermogravimetric analysis of the empty and the loaded nanocarriers.

The vapor pressures of the different active compounds can partly explain the differences in the initial release from the nanocarriers. However, as indicated by the clearly separated instances of mass loss presented in Figure 5, there are several interactions between the active compounds and the nanocarrier. The initial mass loss step is attributed to weakly bound active compounds, i.e., filling of the mesopores. Strongly bound compounds, where the molecules are interacting with the silica surface, will release from the nanocarriers at higher temperatures [53]. The relatively stronger interaction between the polar molecules and hydroxyl groups on the silica surface can be attributed to electrostatic interactions besides hydrogen bonding. This interaction, between the loaded compounds and the silica surface, is an important factor that will determine the retention and the subsequent release profile. 

### 2.4. Stimuli-Activated Release of the Active Compounds

We have previously demonstrated a burst-release of thymol from mesoporous silica nanocarriers in food simulant, with the subsequent volatilization of the compound [34]. However, the release kinetics of encapsulated active compounds in an aqueous solution is dependent on many factors. The physical and chemical properties of the encapsulated compounds, such as the concentration, solubility, vapor pressure and the octanol–water partition coefficient (K_ow_) are of importance, in combination with the properties of the nanocarrier [56]. Thus, the release into liquid media depends largely on the experimental setup. A rapid release might occur into an aqueous solution when the concentration of the loaded nanocarriers is under the solubility level of the specific compounds. Starting with a higher initial concentration of loaded nanocarriers and studying the release as a function of solvent renewal is another technique that gives a more sustained release. Consequently, the level of release can be modulated and should, therefore, be studied at the concentration expected in the final system. Sattary et al. [57] followed the release of essential oils from porous silica particles over several weeks by using a high initial concentration of loaded particles and gradually adding fresh release media. They further demonstrated that the solubility and stability of the essential oils increased with this gradual release into the environment, with a subsequent prolonged antimicrobial effect in their studied soil-plant system.

**Figure 6:**
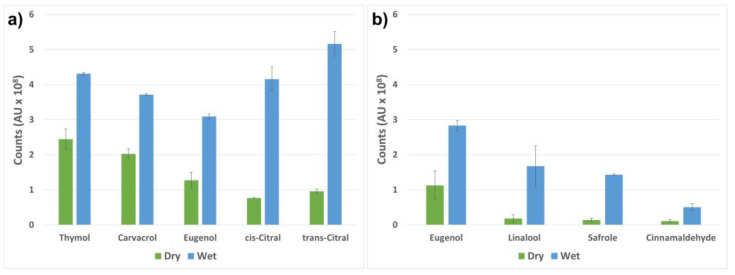
Headspace analysis of the volatile compounds released from the nanocarriers before (dry) and after (wet) activation by buffer solution. The samples were standing for 30 min at room temperature prior to the measurements. The data are presented as the peak areas of the detected compounds vaporized from the nanocarriers. The nanocarriers loaded with pure compounds (thymol, carvacrol, eugenol and citral) are shown in (**a**), while (**b**) presents four of the highest peaks obtained from the nanocarriers loaded with cinnamon oil.

In this study, we investigated the release by analyzing the amount active compounds in the headspace before and after the activation of the release from the loaded nanocarriers. The release was activated by a small amount of buffer solution, which was utilized as a drip-loss simulant. When the buffer was added to the dry hydrophilic nanocarriers, they attracted water molecules, while repelling the loaded hydrophobic compounds. The compounds were further vaporized from the buffer solution and detected in the headspace. Without the activation of the nanocarriers, a significantly lower amount of the active components was detected. The results are presented in Figure 6 as a comparison of the peak areas of the detected volatile compounds vaporized from the nanocarriers with and without the addition of the buffer solution. The largest ratio between the dry and the activated nanocarriers can be seen for the two citral isomers. This can be explained by the vapor pressure, which is the highest for citral (Table 2) among all the tested active compounds. Further, citral has the second highest partition coefficient (log K_ow_), which describes the lipid solubility of the compound. The combination of the hydrophobic character and the high vapor pressure describes the relatively high amount of citral detected in the headspace (Figure 6a). Thymol and carvacrol have similar vapor pressures and log K_ow_ values, which translate into the similar headspace concentrations obtained. The slightly lower concentrations of detected eugenol in the headspace can be explained by the fact that eugenol has a slightly lower vapor pressure and log K_ow_ value compared to thymol and carvacrol (Table 2). The results presented in Figure 6b confirm the possibility of loading pure essential oils, including all the active components, into the silica nanocarriers, with a subsequent activated release. Eugenol, linalool, safrole and cinnamaldehyde were chosen for the demonstration of the activation of nanocarriers loaded with cinnamon oil (Figure 6b). These compounds gave rise to similar discrepancies in headspace concentration after the activation of the nanocarriers. Linalool has the highest vapor pressure, followed by safrole, which together with their relatively high log K_ow_ values result in a large ratio between the dry and the activated nanocarriers. Cinnamaldehyde has a lower vapor pressure and a less hydrophobic character, which may explain its higher retention in the nanocarriers and probable partial dissolution in the buffer.

### 2.5. Antimicrobial Response

The antimicrobial effect of the plant essential oils and their active compounds for food packaging applications has been frequently reported in the literature [58,59,60]. Nonvolatile active components need to be in direct contact with food to instigate antimicrobial activity due to limitations in migration. On the other hand, volatile components can easily release from the carrier and diffuse in the headspace of the packaging. Thus, their antimicrobial activity is less affected by the packaging method and the geometry of the package. 

Contrary to the well-established methods for evaluating the antimicrobial effects of bacteria on solid and liquid media, where the active compounds are in direct contact with the bacteria, there are no standardized techniques for determining the effect of volatile antimicrobials in the vapor phase. For this purpose, the inverted disc volatilization approach (also known as the inverted Petri plate method), as described by López et al. [5], seems to be the most frequently used technique [55,61,62,63]. Even though this method gives a qualitative evaluation, the method does not provide any quantitative information. As a first qualitative assessment of the antimicrobial activity of the loaded nanocarriers in this study, the inverted disc volatilization approach was utilized. For this initial antimicrobial evaluation of the drip-loss activated release, thymol and carvacrol were chosen as the active compounds since these probably are the two most well-studied essential oil components for antimicrobial food packaging applications [64,65,66,67]. Since it is commonly known that gram-positive bacteria are more susceptible to essential oil components than gram-negative bacteria (which is attributed to the external lipopolysaccharide layer of the gram-negative bacteria), the initial experiment was performed with gram-negative *E. coli* to visually evaluate the potential antimicrobial effect. A clear difference in the bacterial growth could be visualized after one day of incubation, which was even more clear after six days of incubation (Supplementary Figure S2). A change in the morphology of the bacteria treated with the active compounds could also be noticed, corresponding to earlier reports by Becerril et al. [11] and Nazzaro et al. [6].

A relatively more recent approach for studying the effect of volatile antimicrobials in the vapor phase is the method reported by Singh et al. [68]. This approach is based on glass vials, where the bottom of the vial contains the volatile compound of interest, and the lid holds a filter paper with the defined inoculum of bacteria. The bacteria are exposed to the volatile compound for a short period of time, and subsequently incubated in liquid media and spread on agar plates to analyze the growth of the bacteria. This method allows for the determination of the total viable counts of the bacteria. An additional advantage of the method is that it can be combined with headspace analysis to monitor the concentration of the active compounds in the headspace. Thus, the same set-up utilized in this study for the GC-MS analysis was used for the antimicrobial evaluation. *B. thermosphacta* and *E. coli* were used as examples of potential food contaminating gram-positive and gram-negative bacteria, respectively.

**Figure 7:**
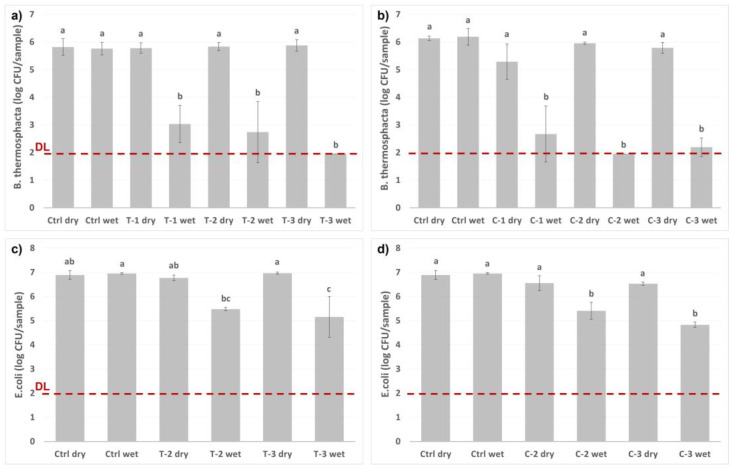
Antimicrobial activity of thymol (T) and carvacrol (C) loaded nanocarriers on *Brochothrix thermosphacta* (**a**,**b**) and *Escherichia coli* (**c**,**d**). Two controls were included: “dry” (empty glass vial) and “wet” (1 mL buffer solution). Two or three different amounts of particles were tested on the bacteria: (1) 4 mg, (2) 8 mg, and (3) 12 mg, either as dry or with the addition of 1 mL buffer solution. The bacteria were incubated at 20 °C for 1 h. One-way analysis of variance (ANOVA), followed by the Tukey test, was performed to analyze the significant differences between the samples. The different letters (a–c) represent the Tukey groups. Samples which do not share the same letter are significantly different (*p* < 0.05). The red line presents the detection limit (DL).

The bacteria were incubated with different amounts of loaded nanocarriers, as presented in Figure 7. The nanocarriers were either in a dry state, or the release from the nanocarriers was initiated by the addition of the buffer solution utilized as a drip-loss simulant. After only one hour of incubation, a ≥4-log reduction in the growth of *B. thermosphacta* was measured, while the dry nanocarriers gave none or a very small inhibitory effect. During the same one-hour incubation period, a 2-log reduction of the *E. coli* was observed when the release of the active compounds was activated.

Many reports have also highlighted the synergistic antimicrobial effects of essential oil components when used in various combinations, with the subsequent benefit of reducing the concentrations needed to effectively inactivate the microorganisms. Consequently, the potential impact on the sensory quality can be kept low. García-García et al. [69] evaluated various mixtures of thymol, carvacrol and eugenol on the inhibition of *Listeria innocua* (a non-pathogenic gram-positive bacterium, closely related to the foodborne pathogen *L. monocytogenes*), and found synergistic effects by using them in binary or ternary mixtures. The positive synergistic antimicrobial effects of thymol and carvacrol have also been reported by others [70,71]. With our investigated system, by loading the volatiles separately into nanocarriers and further potentially applying this as a sachet, different mixtures of the volatiles can easily be adjusted in the package depending on the food product and the specific microflora and packaging system.

## 3. Materials and Methods

### 3.1. Reagents and Materials

All chemicals used for the study were of analytical grade; hexadecyltrimethylammonium bromide (CTAB, ≥98%), tetraethyl orthosilicate (TEOS, ≥98%), cyclohexane (99.5%), thymol (≥99.5%), carvacrol (99%), eugenol (≥98%), citral (≥96%), cinnamon oil (ceylon type), sodium hydroxide (NaOH, ≥99%), ammonium nitrate (NH_4_NO_3_, ≥98%), sodium phosphate monobasic (NaH_2_PO_4_, 98–102%), sodium phosphate dibasic (Na_2_HPO_4_, ≥99%) and halloysite nanotubes (HNT) were purchased from Sigma-Aldrich (Merck KGaA, Darmstadt, Germany). Absolute ethanol was obtained from Antibac (Kiilto Oy, Tampere, Finland).

### 3.2. Synthesis of the Silica Nanocarriers

The silica nanocarriers were of the MCM-41-type, synthesized by the sol-gel method using CTAB as structure-directing agent (SDA), NaOH as a catalyst, and absolute ethanol as a co-solvent to promote a spherical shape. For the synthesis, CTAB and NaOH were completely dissolved in sequence in Milli-Q water under stirring at 45 °C. Ethanol was added, and the stirring continued at 80 °C under reflux. TEOS was used as a silica precursor and added dropwise to the synthesis solution in under five minutes. The reaction was continued for 24 h under vigorous stirring at 80 °C. The molar composition in the synthesis solution was 1 TEOS:0.12 CTAB:0.37 NaOH:73 ethanol:948 water.

The synthesized nanocarriers were collected by centrifugation and the SDA was removed by solvent extraction in NH_4_NO_3_-ethanol solution (molar composition: 1 NH_4_NO_3_:70 ethanol). The extraction process was repeated three times, followed by washing with ethanol to remove the extraction solution. The extracted nanocarriers were dried under vacuum and further stored at room temperature.

### 3.3. Characterization of the Empty Silica Nanocarriers

Scanning electron microscopy (SEM) and transmission electron microscopy (TEM) were utilized to study the size and morphology of the nanocarriers. SEM imaging was performed with a Zeiss EVO 50 microscope (Carl Zeiss, Cambridge, UK) operated at 10 kV. For SEM imaging the dry nanocarriers were placed on carbon tape and further sputter coated with gold-palladium alloy with a Polaron SC 7640 sputter coater (Quorum Technologies Ltd, East Sussex, UK) TEM imaging was performed with a JEOL JEM-2100Plus (JEOL Ltd., Tokyo, Japan) operated at 200 kV, with a camera of type TVIPS TemCam-XF416. Prior to TEM imaging, the nanocarriers were dispersed in ethanol at a concentration of 0.1 mg mL^−1^ and deposited on holey carbon grids.

Dynamic light scattering (DLS) was utilized to evaluate the hydrodynamic size distribution and the dispersibility of the nanocarriers. The nanocarriers were dispersed at a concentration of 1 mg mL^−1^ in Milli-Q water and measured on a Malvern Zetasizer Nano ZS instrument (Malvern Panalytical, Malvern, UK). The Zetasizer instrument was also used for zeta potential measurements to determine the net surface charge of the nanocarriers. A dispersion of 0.5 mg mL^−1^ nanocarriers in 25 mM Hepes buffer at pH 7 was then measured using Malvern’s dip cell kit. Both DLS and zeta potential data were obtained in duplicate, and the data are presented as averages.

Nitrogen physisorption measurements were performed on a BelSorp mini II instrument (MicrotracBEL Corp., Osaka, Japan) at 77 K to determine the specific surface area (SSA), pore volume and pore size distribution (PSD). In each experiment, approximately 70 mg of material was weighed into a 9.001 cm^3^ glass cell. The samples were pre-treated with annealing under a dynamic vacuum, for 2 h at 150 °C. The total SSA was extracted from the nitrogen adsorption isotherms via the Brunauer–Emmett–Teller (BET) method according to the literature [72]. Non-local density functional theory (NLDFT) calculations of PSD were performed using the commercial BELMaster software (MicrotracBEL Corp., Osaka, Japan). The NLDFT calculation method was applied to the adsorption branch using the N_2_ physisorption data collected at −196 °C. For the calculations, a cylindrical pore model was assumed.

### 3.4. Loading of the Active Components into the Nanocarriers

Prior to the loading, the nanocarriers were vacuum-dried for 20 h. The active substances (thymol, carvacrol, eugenol, citral or cinnamon oil) were mixed with the nonpolar solvent cyclohexane and subsequently added to the dried nanocarriers. An excessive amount of the active compound, with a weight ratio of 2:1 (active compound and nanocarrier), was used. The suspension was sonicated and vortexed repeatedly, and further stirred for 24 h. The loaded nanocarriers were separated by centrifugation and washed with cyclohexane with subsequent vacuum-drying to remove the compounds adsorbed on the outer surface.

### 3.5. Determination of the Encapsulated Amount Active Compound by TGA

Simultaneous thermogravimetric analysis (TGA) and differential scanning calorimetry (DSC) experiments were performed in a Netzsch STA 449 F1 instrument (Netzsch, Erich NETZSCH GmbH & Co. Holding KG, Selb, Germany) to determine the loaded amount of volatile compound in the nanocarriers. The loaded nanocarriers were measured in dry, Al_2_O_3_ crucibles and heated from 25 to 900 °C, with a heating rate of 10 °C min^−1^ in an inert atmosphere (100% N_2_ at 50 mL min^−1^). The measurements were baseline corrected using the Proteus software package (Netzsch, Erich NETZSCH GmbH & Co. Holding KG, Selb, Germany).

The method reported earlier [53] was used to calculate the weight percent (wt%) of encapsulated compound in the nanocarriers. Briefly, the weight losses from the loaded nanocarriers were compared to the empty nanocarrier, by both considering the weight loss region up to 120 °C which corresponds to physisorbed water, and the weight loss up to 900 °C which is related to the structural decomposition of the silica matrix, such as the dihydroxylation of the silica network. Thus, the residual fraction (RF) of the empty silica nanocarrier was determined by dividing the percent mass residue (MR) at 900 °C by the MR after water loss at 120 °C (as shown in Supplementary Figure S3), i.e., according to the calculation:

$$RF\left(empty\right)=\frac{MR_{900}\left(empty\right)}{MR_{120}\left(loaded\right)}$$

Further, for all the five loaded silica nanocarriers (SNC) the following calculations were used to determine the wt% encapsulated compound per whole SNC:

$$RF\left(loaded\right)=\frac{MR_{900}\left(loaded\right)}{RF\left(empty\right)}$$

$$wt\%\;encapsulated\;compound=\frac{MR_{120}\left(loaded\right)-RF\left(loaded\right)}{MR_{120}\left(loaded\right)}\times 100$$

### 3.6. Determination of the Retention of the Encapsulated Active Compounds in Ambient Conditions 

Ultraviolet-visible (UV-Vis) measurements were performed for evaluating the retention of the active compounds loaded into the nanocarriers in ambient conditions. The retention was determined by weighing a known amount of nanocarriers into small weighing cups and leaving these open in ambient conditions for various time points (1, 3, 7 and 9 weeks). Two separate samples were prepared for each time point. At the defined time points the nanocarriers were collected, and a known amount of ethanol was added. The remaining active compounds were dissolved by sonication and shaking. The nanocarriers were separated by centrifugation and the supernatants were measured in duplicate on a Spectrostar Nano Ultraviolet-visible (UV-Vis) spectrophotometer (BMG Labtech, Ortenberg, Germany) at the specific wavelengths for the compounds. The wavelengths were selected based on the highest adsorption of the specific compounds dissolved in ethanol, as follows: citral 240 nm, thymol 279 nm, carvacrol 280 nm, cinnamon oil 282 nm, and eugenol 283 nm. The loading degree of the volatile compounds left in the nanocarriers was assessed by using a standard curve made in ethanol at specific wavelengths. The data are presented as an average of four measurements (two separate samples measured in duplicate). 

### 3.7. Evaluation of the Stimuli-Activated Release of the Active Components by Simulated Drip-Loss

For headspace analysis of the volatilized active compounds from the loaded nanocarriers, both in dry conditions and after the addition of simulated drip-loss, gas chromatography–mass spectrometry (GC-MS) was utilized. To simulate drip-loss from chicken a 0.1 M phosphate buffer with a pH of 6 was prepared and used to study the activated release of the volatile compounds. The release was assessed by comparing the amount of active volatile compounds in the headspace before and after the addition of the buffer solution to the dry loaded nanocarriers. 

For the measurements, 1 mg of loaded nanocarriers were weighed into 20 mL glass vials and tightened with a screw cap with Teflon sealing. The vials were either directly sealed or a small amount of food simulant (1 mL) was added to the dry nanocarriers prior to sealing the vials. The vials were kept at room temperature for 30 min to allow the active compounds to vaporize. An automated Gerstel MPS 2 system (Gerstel GmbH & Co.KG, Mülheim an der Ruhr, Germany) with a dynamic headspace sampling system integrated with an Agilent 7890B gas chromatograph, GC (Santa Clara, CA, United States) and thermal desorption unit interfaced with the GC injector port was used for the analysis of the compounds. The vaporized compounds were purged and trapped at room temperature on activated charcoal (Tenax GR, mesh size 60/80, Alltech Associates Inc., Deerfield, IL, USA) sorption tubes. An Agilent 5977B mass spectrometer (Santa Clara, CA, United States) interfaced with the GC, operating in EI mode (70 eV), was used as a detector. The GC temperature program was as follows: 30 °C (0.5 min hold time)−1 °C/min to 40 °C−3 °C/min to 70 °C−6.5 °C/min to 230 °C (5 min hold time). Agilent Mass Hunter software (Santa Clara, CA, United States) was used to process the acquired measurement data and integration of peak area. The chromatographic peaks from the vaporized active compounds were analyzed with respect to the differences in peak area between the compounds vaporized from dry particles and particles dispersed in food simulant. All tests were performed in duplicate, and the results are presented as averages with standard deviation.

### 3.8. Determination of the Antimicrobial Response of the Released Active Compounds

The antimicrobial response of the stimuli-activated release of the active compounds from the nanocarriers was evaluated based on the growth of the foodborne *Brochothrix thermosphacta* strain MF6860 (own collection; isolated from chicken fillet) and *Escherichia coli* ATCC 11229, essentially by the method reported by Singh [68]. For this evaluation thymol- and carvacrol-loaded nanocarriers were chosen. In short, specific amounts of loaded nanocarriers were weighed into glass vials. The same 20 mL glass vials used for the GC-MS analysis were utilized here. As for the GC-MS measurements, the vials were either directly sealed or a small amount of food simulant (1 mL) was added to the dry nanocarriers prior to sealing the vials. The vials were further incubated at room temperature for 30 min to allow the active compounds to vaporize. Whatman No. 1 filter paper discs were cut to the size of the rim of the vial and placed in identical lids as used for the vials. The bacteria were grown in tryptone soya broth (Oxoid, Basingstoke, UK), at 37 °C (*E. coli*) or 25 °C (*B. thermosphacta*), to prepare suspensions containing 1 × 10^9^ colony-forming units (CFU)/mL. The filter papers placed in the lids were impregnated with 10 µL of bacterial suspensions whereafter the vials containing the nanocarriers were swiftly opened and the lids were replaced with the ones containing the bacteria suspension. The replacement of the lids was performed swiftly to minimize vapor loss from the vials. The vials were incubated at 20 °C. After one hour of incubation, the vials were opened, and the filter discs were removed and placed in tubes containing 4.5 mL peptone water. The tubes were mixed to remove all the bacteria from the filter disc, whereafter the bacteria suspension was further diluted and plated on agar plates (Tryptone Soya Agar) by The Whitley Automated Spiral Plater, WASP (Don Whitley Scientific Ltd., Shipley, UK). The viable bacteria number after incubation at 37 °C for 24 h (*E. coli*) or at 25 °C for 24–48 h (*B. thermosphacta*) was determined by counting the CFUs. The experiments were performed in duplicate, and the results are presented as averages with standard deviation, and statistically analyzed by one-way ANOVA and the Tukey test with a 95% confidence level.

## 4. Conclusions

The delivery of antimicrobials in a controlled manner into food packages is important for maintaining food safety and quality. The trend is going towards greener and more environment-friendly packaging materials, often with poorer barrier properties which are not suitable for traditional packaging with a modified atmosphere. Thus, dual-action sachets with a prolonged and controlled release of antimicrobial compounds can sustain the antimicrobial stress on the targeted bacteria over the required shelf-life period. For these eco-friendly food packaging applications, natural antimicrobials, such as essential oils are increasingly studied. However, due to the volatility of these compounds and their tendency to oxidize, a suitable carrier matrix is required. Porous silica particles are well-suited for food packaging applications due to both their vast design options and their biocompatibility. Both silica nanoparticles and the by-product silicic acid are approved by the EU commission regulation No 10/2011. This is of importance not only for the safety when incorporated into the food package, but also with regard to the potential release of nanoparticles into the environment after the disposal of the packaging materials. It must be mentioned that most of these antimicrobial volatile compounds are odor active and might affect the flavor of the food product, and some of them have low odor thresholds, so for practical applications, it should be documented also that this shelf-life strategy does not affect the sensory quality of the food. 

In the present study, mesoporous silica nanocarriers have been synthesized with a special focus on the incorporation of the volatile compounds. The high surface area and optimal pore structure of these nanocarriers enabled a high loading capacity with a subsequent prolonged vaporization rate of the compounds which flattened out after several weeks. Through the addition of simulated drip-loss the release of the compounds was activated and gave a clear antimicrobial effect, which was demonstrated on the food spoilage bacteria *B. thermosphacta* and the potentially pathogenic bacteria *E. coli*. Consequently, we would expect an increased availability and subsequent antimicrobial effect of the natural compounds for active food packaging by using the stabilizing nanocarriers with the prolonged-release which is activated by the food product itself.

## Figures and Tables

**Table 1:** Amount active compounds encapsulated in the silica nanocarriers (SNC) per whole mass of the loaded nanocarrier or per mass of empty nanocarrier.

Compound	wt% Compound/Whole SNC	wt% Compound/empty SNC
Thymol	27	37
Carvacrol	26	35
Eugenol	29	41
Cinnamon oil	26	35
Citral	34	52

**Table 2:** Physico-chemical properties of the active compounds obtained from [54,55].

Compound	Vapor Pressure at 25 °C [mm Hg]	log K_ow_
Thymol	0.038	3.30
Carvacrol	0.030	3.49
Eugenol	0.022	2.27
Citral	0.091	3.45
Linalool	0.160	2.80
Safrole	0.071	3.45
Cinnamaldehyde	0.029	1.90

## Data Availability

The data presented in this study are available on request from the corresponding authors.

## Supplementary Materials

The supporting information can be downloaded at: https://www.mdpi.com/article/10.3390/ijms23137032/s1.
