# Supplementary material for: Controlled Release of Volatile Antimicrobial Compounds from Mesoporous Silica Nanocarriers for Active Food Packaging Applications
Source: Int J Mol Sci. 2022 Jun 24;23(13):7032. doi: 10.3390/ijms23137032 (PMC9266657; doi:10.3390/ijms23137032)
Supplement: Supplementary file 1 [file ijms-23-07032-s001.zip › ijms-1762672-supplementary.pdf]

Supplementary data to: Controlled release of volatile antimicrobial compounds from mesoporous silica nanocarriers for active food packaging applications

Tina Gulin-Sarfraz*, Georgios Kalantzopoulos, John-Erik Haugen, Lars Axelsson, Hilde Raanaas Kolstad, Jawad Sarfraz*

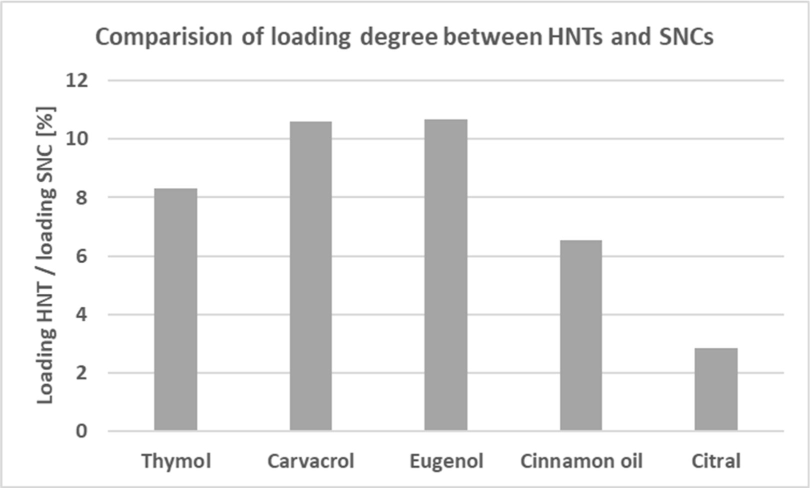

Supplementary Figure S1. The total amount of the encapsulated active compounds in halloysite nanotubes (HNTs) are compared with silica nanocarriers (SNCs) and are presented in percentage.

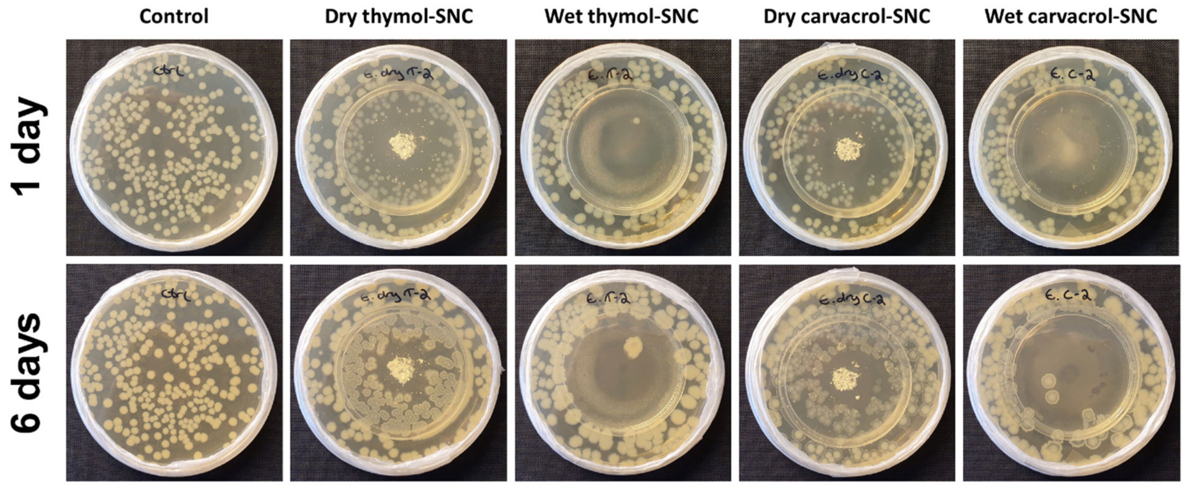

Supplementary Figure S2. Antimicrobial effect of silica nanocarriers (SNC) loaded with thymol or carvacrol on Escherichia coli. The pictures were taken after one day and six days of incubation at 37 °C. A clear effect on the growth of the bacteria can be seen for both thymol and carvacrol loaded SNCs. However, a more pronounced antimicrobial effect is revealed after activating the release from the SNCs (named “wet” in the figure). A change in the morphology of the bacteria treated with the active compounds could also be noticed.

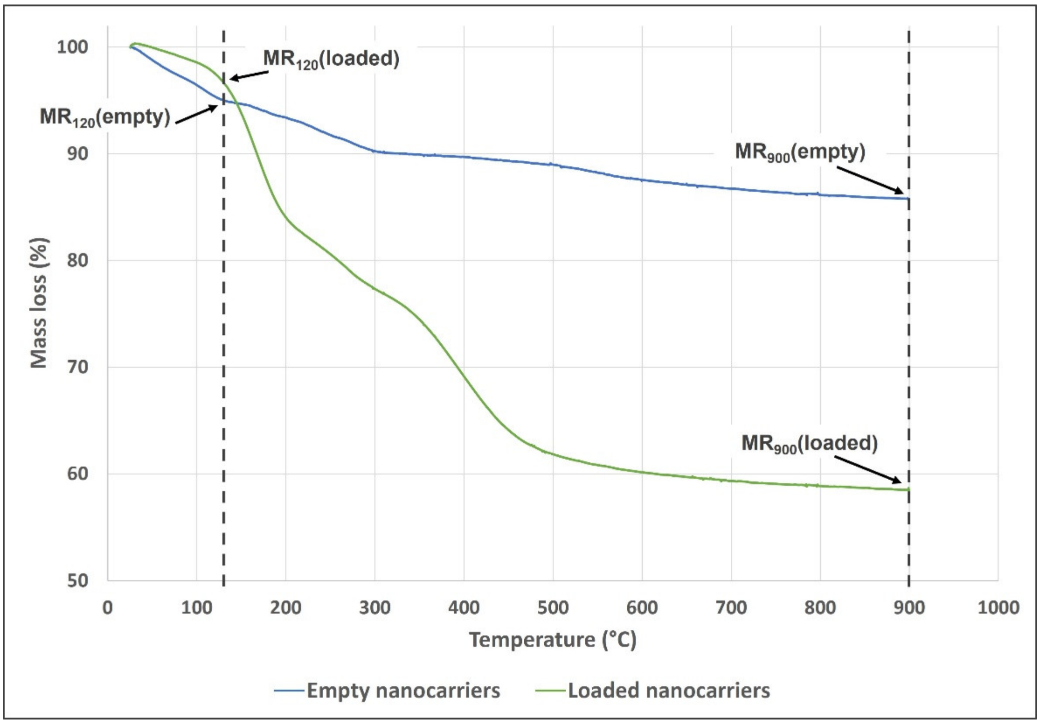

Supplementary Figure S3. Examples of percent mass residue (MR) values are illustrated to assist the explanation of the equations used for determining the encapsulated amount active compound by thermogravimetric analysis (TGA).
